# “It Was Part of the Plan, so I Showed up”: An Exploration of Patient Experiences With an Embedded Exercise Referral Process During Cancer Treatment

**DOI:** 10.1177/10732748261451084

**Published:** 2026-05-12

**Authors:** Mary A Kennedy, Jack Dalla Via, Kim Edmunds, Pam Eldridge, Yvonne Zissiadis, Lauren Fortington

**Affiliations:** 1 Nutrition & Health Innovation Research Institute, School of Medical and Health Sciences, 204605 Edith Cowan University, Perth, WA, Australia; 2 Institute for Physical Activity and Nutrition, School of Exercise and Nutrition Sciences, 549584 Deakin University, Geelong, VIC, Australia; 3 Centre for the Business and Economics of Health, 589079 University of Queensland, Brisbane, QLD, Australia; 4 Radiation Oncology, 366577 GenesisCare, Perth, WA, Australia; 5 Exercise Medicine Research Institute, School of Medical and Health Sciences, 204605 Edith Cowan University, Perth, WA, Australia

**Keywords:** exercise, radiation oncology, medical oncology

## Abstract

**Introduction:**

Exercise is a key part of cancer care, yet its integration into routine practice remains limited. This study explored the experiences of people living with and beyond cancer, with an updated, opt-out referral model embedded within a supportive oncology setting, focusing on initial engagement with exercise physiology appointments. Through this model, patients were automatically scheduled for a consultation with an in-house exercise professional during their cancer treatment.

**Methods:**

A qualitative descriptive design comprised of two focus groups with 13 participants was employed to explore views across pre-defined elements of the model: referral to exercise, appointments, counselling, and tailoring.

**Results:**

Participants valued the automatic appointment structure as it normalised exercise as being part of their treatment and reduced the burden from decision-making and arrangement of appointments. Trust in their treating doctors and nurses, together with consistent messaging, reinforced exercise as a medically endorsed intervention. Counselling on exercise addressed misinformation, highlighted the physiological rationale, and helped foster a sense of control and agency. The inclusive gym environment and tailored plans further supported engagement with exercise, from which the importance of collaborative but individualised care was noted. While the opt-out process facilitated initial uptake, sustained access to exercise services beyond the clinical setting remains a challenge.

**Conclusions:**

Findings support the feasibility and acceptability of integrated referral pathways and underscore the need for system-level changes and community partnerships to ensure long-term, equitable access to exercise in cancer care.

## Plain Language Summary

Why we did this study: Exercise helps people with cancer, but most don't get referred to exercise services. We tested a new system where cancer patients automatically got an appointment with an exercise professional during their treatment, without having to ask for it. What we did: We talked to thirteen people with cancer in two group discussions to understand what they thought about this automatic referral system. What we found: People liked getting an automatic appointment because: It made exercise feel like a normal part of their cancer treatment. They did not have to make decisions or organise appointments themselves when they were already overwhelmed. When their doctors and nurses talked about exercise, it felt like proper medical advice they should follow. The exercise professional helped clear up wrong information and explained how exercise actually helps their body. Having personalised exercise plans that matched their individual needs made them feel more in control. The gym felt welcoming and inclusive. The main challenge was keeping up with exercise after their cancer treatment finished and they no longer had access to the hospital's exercise services. What this means: Automatically giving cancer patients exercise appointments works well and people like it. However, hospitals need to work with community gyms and exercise providers so people can continue exercising after their treatment ends.

## Introduction

Exercise is a critical part of cancer treatment, shown to reduce treatment-related side effects and improve quality of life and health outcomes.^1,2^ In response, many professional bodies recommend integrating exercise into routine care.^3-6^ Guidelines advocate for individualised exercise prescriptions incorporating both aerobic and resistance training, tailored to a person’s treatment phase, functional capacity, and comorbidities. To achieve this level of care, clinicians are encouraged to initiate discussions with their patients about the role of exercise in cancer recovery, recommend adherence to evidence-based exercise guidelines, and refer patients to qualified exercise professionals. Despite these recommendations, persistent barriers impede routine integration into oncology care and engagement in exercise among cancer survivors is generally poor.^7-9^

Implementation barriers impact provision of exercise at patient, provider, organisational, and systemic levels of care.^10^ For example, patients face side effects, emotional distress, low motivation, limited awareness, logistical and financial challenges, and health literacy issues. Providers may lack guideline knowledge, have safety concerns, or time constraints. Organisational gaps in infrastructure and coordination hinder delivery, while systemic issues, like absent mandates and funding, limit integration. Evidence-based implementation strategies remain a much needed exercise into routine care.

Several studies have explored strategies to overcome barriers to exercise integration in cancer care. Key behaviours that support successful implementation include identifying suitable patients, facilitating referrals, and providing ongoing support.^11^ When conversations about exercise are initiated by oncologists, patients are more likely to take action.^12^ However, ambiguity around referral responsibility suggest a need for clearer structural processes.^12^ Additionally, co-designed interventions have been reported to improve clinician confidence and increase referral rates when supported by streamlined processes.^13^ Drawing strong conclusions is challenging due to varying definitions, frameworks, healthcare contexts, and focus across care phases. Nevertheless, three consistent factors stand out in support of successful referral to exercise in cancer care: (1) the central role of clinicians in promoting exercise, (2) the need for tailored exercise support, and (3) system-level changes to ensure long-term success.

Our research team has an ongoing partnership with a private cancer care organisation in Western Australia. Initially, only 12% of eligible patients attended an exercise program that was co-located at one site of the care organisation.^14^ To address low patient reach, we implemented two key changes to the referral model. Firstly, the referral process was integrated into the medical workflow system and oncologists were provided with clear instructions on how to initiate the referral. In turn, this enabled an opt-out approach (termed ‘co-located exercise service (CoLEC) integrated workflow’), automatically flagging clinically eligible patients to the booking team for arrangement of an exercise physiology appointment.^15^ These changes increased reach to 32%.^15^ Nonetheless, substantial gaps in access remained, highlighting the need for further refinement to achieve guideline-concordant care in exercise oncology.

This study explores perspectives on the updated referral process, aiming to understand the information provided about exercise and experiences of being referred to an exercise appointment. The focus is on the process up to and including the initial appointment with the exercise service, rather than subsequent exercise prescription, delivery or outcomes. Insights gained are intended to inform the development of future strategies for embedding exercise referral practices into routine oncology care.

## Methods

A descriptive qualitative design was employed to gain practical insights about the referral process from the participants’ perspectives.^16^ This design was chosen to explore the experiences of people with cancer within the specific clinical and organisational context being explored, with the aim of informing and enhancing the exercise referral workflow. Focus groups were selected because they were well-suited to capture how a standardised, shared referral pathway was perceived across patients with diverse cancer diagnoses and treatment histories, enabling collective sense-making whereby participants could validate, build on, and nuance each other’s experiences in ways that individual interviews would not have afforded. The research was approved by the Edith Cowan University Human Research Ethics Committee (2023-04765-KENNEDY). Participants had opted to receive information about potential research projects through their care organisation, responded to an email outlining this study, and provided their consent to take part in the focus group.

### Setting

**Figure 1.**
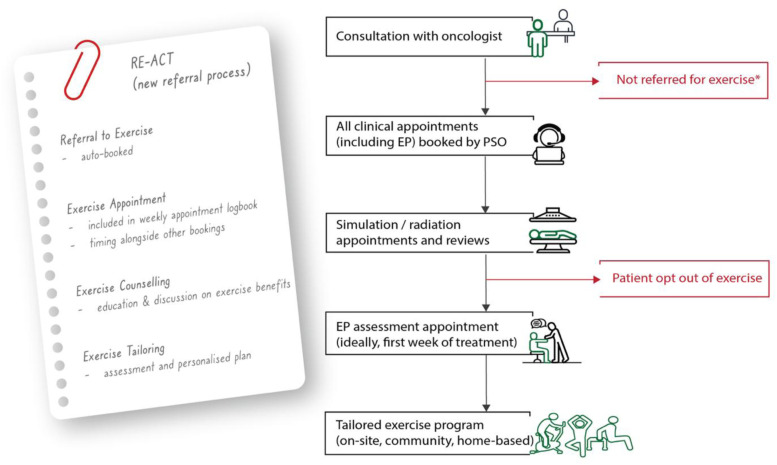
Referring to exercise: flow chart of new opt-out process. *Inpatients and some short course patients are not currently referred due to limits with capacity; EP = exercise physiologist; PSO = patient service officer

Several public and private cancer treatment centres are available in Western Australia. In this study, our team worked with one private care organisation at two of their treatment sites. In 2023-2024, a revised process for referrals to exercise was trialled in the partner organisation. This involved modifying the earlier CoLEC integrated workflow^15^ to the Referring to Exercise: Appointment, Counselling, Tailoring (RE-ACT, Figure 1).

The referral process was designed to align with the Clinical Oncology Society of Australia (COSA) 2018 position statement recommending that all people diagnosed with cancer receive a discussion, recommendation, and referral to exercise.^4^ During their medical intake, all new patients scheduled for radiotherapy (excluding short course and inpatient) are automatically booked into a consultation appointment with an accredited exercise physiologist (EP), by a patient service officer (PSO, staff member who provides administrative and booking support). Short course radiotherapy patients and inpatients were excluded due to the limited treatment timeframe and logistical constraints respectively. No additional clinical criteria were applied by oncologists or exercise professionals to determine eligibility for referral.

The EP appointment is included in the patient’s logbook, which details all upcoming clinical activities, including radiation, oncologist appointments and nursing team reviews. On arrival to the care centre, patients scan into their first appointment of the day with their logbook, and team members guide them to each subsequent appointment location. The inclusion of exercise appointments in this process was intended to reinforce the service as part of usual care.

A 45-minute initial EP consultation covers education regarding the benefits of exercise as well as patient history (oncology, medical, exercise) and preferences. Subsequently, the EP will provide recommendations based on the patient’s needs and preferences, which might include continued on-site appointments with the EP, connection to local community exercise or development of a home or gym-based program.

### Study Recruitment

Participants were recruited through the partner organisation. As part of the standard treatment consent process, all patients are given the option to allow their data to be used for research purposes. The PSO identified individuals who had attended at least one session at one of two participating sites within the relevant timeframe and distributed an email invitation to all eligible participants. Eligibility criteria included a confirmed diagnosis of cancer and completion of active treatment involving radiation therapy within the last 6 months. This purposive sampling strategy ensured that participants had direct experience with the referral process under investigation.

### Consent and Participation

Interested participants responded directly to the research team following the email invitation. They were provided with a plain language statement outlining the study purpose, procedures, and ethical considerations. Focus group attendance was arranged with those who consented to participate. All participants provided written informed consent prior to any data collection. Participation was entirely voluntary and had no impact on access to treatment, care, or exercise services.

### Data Collection

#### Participant Information Questionnaire

Participants completed a brief questionnaire on paper before attending the focus group (available in Supplementary file 1), comprising demographic information (e.g., age, gender, marital status, education, occupation) and a mix of closed (multiple choice) and open text questions relating to their physical activity behaviours prior to, during, and following cancer treatment. Questionnaire responses are used only to describe the participants (i.e. we do not analyse their open text answers in this study).

#### Focus Groups

Two in-person focus groups (Group 1= 78 minutes; Group 2 = 98 minutes) were conducted in a meeting room on a university campus. After initial information on introductions (i.e. facilitator did not know the topic area or clinic staff), housekeeping (i.e. location of toilets, emergency alarm sounds) and focus group participation (i.e. speaking in turn, not sharing personal discussion) the groups were directed to three topics.

• Understanding of, and engagement with, exercise before diagnosis
• Communication about exercise between the diagnosis and first appointment with the exercise team
• The information received from the exercise team, and other care team members.

Where prompts were needed, participants were asked to think about what was most useful, what was missing, what was surprising or what made things ‘click’ (make sense) for them. A visual was used to support the discussion to keep the topic in focus, and to help shift the conversation to the next topic, when required (Supplementary file 2). All participants were given the opportunity to respond to each question. The discussions were audio recorded, and field notes were written. There were three research members in attendance at each group: a research assistant who had supported the participants’ attendance (e.g. questionnaire completion, parking/logistics, refreshments), the focus group facilitator (author LF) and the lead researcher (MAK). Facilitator LF is a PhD trained research academic with experience conducting focus groups of a similar nature. She was independent from all participants, the referral process and care team, and solely asked questions for the data collection. Lead researcher MAK is PhD trained and was available to introduce the session and clarify any question or issues that arose relating to the referral process, following the discussion.

### Data Analysis

Data collected from the questionnaires was tabulated and presented descriptively to provide an overview of the participants, while being careful to protect individual privacy. Focus group audio recordings were transcribed verbatim by a professional transcription service. To protect participant confidentiality, identifying details were removed (e.g. names replaced with pseudonyms; specific place or care providers replaced with a description e.g. physio, metropolitan clinic). Each transcript was reviewed multiple times to ensure familiarity with the content and context of participant responses. With only two transcripts to manage, no specialist software was used, the data were managed in Microsoft Word. Relevant excerpts were highlighted, and similar ideas were grouped together by author LF. These groupings were then refined and organised within broader thematic categories, ensuring that subthemes were derived directly from the data and authentically reflected participants’ experiences. Representative quotes, edited for readability, are presented throughout the results to illustrate and support the findings. Throughout the analysis, the authors maintained a reflexive stance, acknowledging their influence on the findings, which initiated from the design of the referral process (MAK, YZ, PA), the development of the focus group questions (MAK, LF), through to the interpretation and finalisation of results (all authors). The approach provided structure to the research while allowing flexibility to capture unexpected insights and learnings from participants. Rigour, particularly confirmability (i.e. the results were derived from the data and not the researchers’ biases or interpretations), was addressed in multiple ways, but emphasised peer debriefing, member checking and transparency in the development of subthemes. 1^17^ The research team reviewed and discussed the subthemes, critically considering the findings and where the findings best fit in the referral process. This process continued iteratively until all authors were satisfied that the results accurately reflected the participant feedback.

An additional step in the analysis was to review results using Microsoft CoPilot (with Enterprise Data Protection), essentially serving as an additional author for validation (i.e. member checking).^18^ Transcripts were uploaded, with prompts directing that the results be queried within each section to explore whether there were any further codes relevant to the study aims that had not been identified. Additional quotes were identified, and wording ideas for subthemes, but no additional results (ideas for subthemes) were included.

## Results

### Groups and Participants

Table 1 presents insights on participants in each group (sex, employment, education, marital status and physical activity). Group 1 comprised 6 people (5 men and 1 woman), while group 2 comprised 7 people (2 men and 5 women). The median age was 73 years (range 51-80, mean 69). All patients had received radiation therapy (median treatment sessions = 20, range 4-39, mean 23). Six people had prostate cancer, five had breast cancer and two had skin cancer/melanoma. Additional to the primary cancer, two people also had metastases (lung or bone). Half of the participants in each group self-reported meeting physical activity guidelines pre-cancer diagnosis. Most participants were no longer working; four noted part time, volunteer or occasional work.

**Table 1.** Summary of Participant Information for Each Focus Group (n=13 in 2 Groups)

Group	ID	Gender	Employment/Activity	Highest education	Met physical activity guidelines pre-cancer
Group 1	1	Man	Retired	Unclear	Yes
Group 1	2	Man	Retired	High school	/
Group 1	3	Woman	Working part-time	Diploma/Certificate	Yes
Group 1	4	Man	Retired	Diploma/Certificate	No
Group 1	5	Man	Retired	Trade or technical qualification	Yes
Group 1	6	Man	Retired	Some high school	No
Group 2	7	Woman	Full-time home duties	Bachelor’s degree	No
Group 2	8	Man	Retired	Bachelor’s degree	Yes
Group 2	9	Woman	/	/	/
Group 2	10	Woman	Retired	Diploma/Certificate	Yes
Group 2	11	Woman	Working part-time	Bachelor’s degree	No
Group 2	12	Man	Retired	Diploma/Certificate	No
Group 2	13	Woman	Full-time home duties/retired	Some high school	Yes

/= missing information.

### Referral phases and subthemes from focus groups

**Figure 2.**
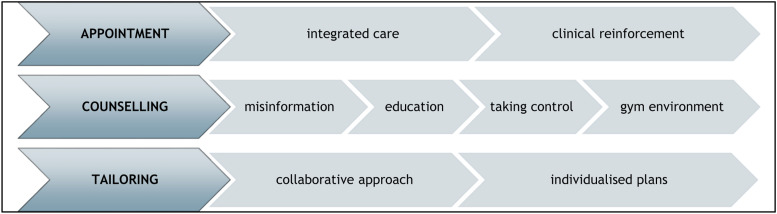
The three referral phases, each with two to four subthemes identified in each

Within the three phases of the referral process, eight subthemes are described (Figure 2).

### Phase 1: Appointments

Findings identified two avenues from which patients engaged with their initial exercise appointment: integrated care; and clinical reinforcement.

#### Subtheme 1.1 Integrated Care

“An appointment was made, thou shalt turn up....”

Having exercise appointments booked automatically and included within the appointment logbook was a major factor in participation; it meant patients did not question attendance. Participants consistently emphasised that exercise should be a standard, integrated part of the treatment pathway, not an optional extra. The integrated appointment helped participants initially see exercise as a normal and expected part of care. The appointment structure removed the burden of decision-making and reinforced the legitimacy of exercise as part of care.

“The nurse told me to go. I had no choice.”

“They just slotted you in... it’s almost like they force you, which is good.”

“You had the appointment with the nurse, radiation, physio, and gym. You didn’t question it. You just went.”

#### Subtheme 1.2 Clinical Reinforcement

“They hammered it home - it's top of their consultation. They don't say it once. It's over and over.”

Trust in clinicians and consistent messaging across the care team were powerful motivators. Exercise was framed as a medically endorsed treatment, not a lifestyle choice. Early and consistent messaging from healthcare providers-oncologists, nurses, physiologists-was crucial. The inclusion of exercise in the printed weekly logbook reinforced its importance, placing it alongside radiation and hormone therapy.

### Phase 2: Counselling

Once patients attended their initial appointment, exercise counselling offered an opportunity to deepen engagement. Four subthemes were identified that reinforced the value of exercise: addressing misinformation; education on the benefits of exercise; taking control; and addressing barriers to getting started.

#### Subtheme 2.1 Addressing Misinformation

“When they first mentioned exercise, I was a bit wary… it’s like a myth. You need to rest. You need to recover.”

Many participants initially believed that a cancer diagnosis necessitated complete rest and withdrawal from physical activity. This misconception, rooted in fear and outdated beliefs, led to hesitation and inactivity.

“You’re diagnosed with cancer. You need to stop everything. Your life kind of stopped.”

“My mum said I needed to rest more, but I found that exercise helped boost my levels and maintain them. That, for me, was a turning point.”

Through educational resources, supportive conversations with clinicians, and peer experiences, these myths were gradually dispelled. Participants began to understand that exercise could be safe, beneficial, and even essential during treatment and recovery. This transformation highlights the importance of clear, evidence-based messaging to counteract misinformation and empower individuals to take an active role in their recovery.

#### Subtheme 2.2 Education on Benefits of Exercise

“I didn’t have a clue [exercise] would be an additional benefit…it was really quite stunning for me to find that out.”

Educational content that linked exercise with the clinical treatment of cancer, especially videos and podcasts, played a pivotal role in shifting perceptions about exercise during cancer treatment. Participants began to see exercise not as a ‘nice to have’, but as a biologically active intervention. Understanding the mechanisms behind the role of exercise in cancer treatment increased motivation and engagement. When patients grasped how exercise could enhance outcomes, they were more likely to participate.

“The message I got was it helps with your symptoms of radiation... I never got fatigued, at all.”

“In that video they talk about gym exercises, weight-bearing stuff... how blood sends a message to your immune system to combat the cancer. It helps eat the cancer away, in a way.”

“My second chat was with the exercise physiologist who explained immunity and exercise.”

#### Subtheme 2.3 Taking Control

“It was a mental thing. I wasn’t in control of anything else, but that’s one thing I could do.”

Participants often described a moment of emotional clarity, whether frustration, fear, or determination, that drove them toward action with exercise, in part because it was seen as a way to regain control during a vulnerable time. The counselling phase of the exercise appointment helped patients connect exercise with personal meaning and resilience, reinforcing it as a proactive choice in their care journey.

“I have a very big thing that I need to be part of my care. If we’re doing a drug treatment, I need to know what it is and how it works and work together so that I feel like I’ve got control. That’s my main thing. Yeah.”

To maximise impact, participants recommended clearly communicating the holistic benefits of exercise, specifically noting enhanced immunity, improved self-perception, and emotional uplift, rather than relying solely on information materials.

“Highlight the benefits… not just, ‘here’s a leaflet.’”

For most participants, the structured appointment process (described earlier) was well accepted but for a small number of participants, the counselling phase and education provided was a way to re-gain control of what had otherwise felt to them like an automated care journey.

#### Subtheme 2.4 gym Environment

“Do I belong here? That was my first thought. Not being a gym person.”

For many, the concept of entering a gym was intimidating and unfamiliar and one of the most frequently cited obstacles to engaging in exercise. Traditional gyms were described as unwelcoming spaces, dominated by loud music, fit bodies, and a culture that felt exclusive.

“I’ve walked past a lot of gyms but never gone in… They’re full of sweaty bodies, pumping away with loud music and I don’t look good in a leotard.”

“You sit there trying to do it, and some muscley guy comes up and says, ‘You just blocked me.’ I’m not trying to take gym selfies—I just want to know I’m doing the right thing.”

In contrast, the exercise setting at the partner organisation was described as inclusive and welcoming, helping participants feel more comfortable and open to engaging. Counselling played a role in acknowledging these concerns and offering reassurance and fostering a collaborative approach. The EPs helped participants feel seen, supported, and empowered.

“I think that motivation to actually go to a place and to have it whereby it’s not a huge, big gym – whereby it’s a contained sort of thing. You don’t see all these young people that have got fantastic bodies and are doing all this exercise. At [Partner organisation], we’re all very similar and different shapes, and in our ability.”

“Having someone say, ‘These are the steps we can take,’ and working together—that’s a big thing. If it felt like, ‘Here’s a bike, go do it,’ I wouldn’t have done it.”

### Phase 3: Tailoring

The personalisation of exercise programs was described in two subthemes: collaborative and individualised. Participants appreciated that the EP listened to their histories and adapted education and exercises to their specific needs (e.g., lymphedema, chronic pain, post-surgery limitations).

#### Subtheme 3.1 Collaborative Approach

Participants consistently emphasised the value of individualised exercise discussions that respected their personal history, physical limitations, and preferences, and fostered a sense of autonomy and emotional support, enhancing engagement and motivation. Encouragement from program facilitators played a pivotal role. This sense of shared commitment was deeply appreciated. Participants also reported feeling empowered finding out what they were still capable of through the EPs support.

“What do you want to do? Where do you want to go? How do we get you there? Do you want to be in a gym? No. Do you have this at home? Yes. So you set that program and that’s great. But for somebody to turn around – I would find it very difficult for somebody to say, you need to do this, you’ve got to help yourself […] Because it’s like – it’s not about pushing somebody to do it, it’s about encouraging somebody to do it, to their limit.”

“It was lovely. I said, look, I can’t even move my leg. She said, well, get on the exercise bike. I said, I can’t do that. She said, there’s a reclining exercise bike. Sat on that and I can do it. I was empowered straight away that I could do something I didn’t think I could.”

#### Subtheme 3.2 Individualised Plans

Tailoring also considered other challenges the patients were managing including carer responsibilities (children, parents and/or spouse), work and health diagnoses for them and their loved ones. These were not insignificant issues to the participants with their reflections demonstrating shared feelings of anguish and compassion amongst the group.

“Asking for history, for me, is very important - that they understand my history because I’m unique. They’ve made a unique exercise program for me”

“I needed to do the exercising because this is the only way I’m going to get better, and I have to get better to look after him.”

“I found that they tailored what they gave me. I don’t see a lot of people in the gym doing the things that I do so I figured that [EP] has tailored it for my back, which I’ve had chronic issues with. Instead of pulling the bar down this way, I pull it down this way. It’s just tailoring different things. I found that really personalised. I found that really good. I appreciated it because she had heard me. In our first meeting, she had heard. She listened to me.”

### Opportunities to Improve

Outside of the main analysis, transcripts were also considered specifically to identify further opportunities to improve the exercise referral and engagement process. One area for consideration is when the most beneficial timing of when exercise information should be provided and when the initial exercise appointment should take place. For these participants, there was no consensus as to the most beneficial timing of when exercise information should be provided. Most participants felt the timing alongside simulation and other appointments was ideal, while a small number wished for earlier access to exercise, at the point of diagnosis.

“I felt like it waited too long. I wanted it almost very quickly.”

The absence of structured follow-up left some participants feeling unsupported and uncertain about how to manage exercise throughout their cancer treatment. Participants felt that integration into long-term care plans and consistent follow-up could reinforce the importance of exercise and sustain engagement.

“At the start, you get a lot of focus as you get your program set out. But then from then on, you’re left to your own motivation.”

“Even a closing one to actually motivate me. Like I’m one that has not continued. That last appointment, I had the – I did the bike before I had my treatment. I haven’t been back since. I haven’t received any – that was it. Rang the bell and there’s been no appointment. If someone had said, oh, I haven’t seen you in a while. You do have three months, you know, free gym membership post treatment, it would have been like, ooh, yeah…”

## Discussion

Exercise has consistently been shown to be feasible and beneficial for cancer patients^19,20^; the current challenge lies in making it a routine part of oncology care. This study explored patient experiences with an integrated, opt-out referral process, focusing on initial engagement with the exercise appointment and counselling tailored to patient needs. Participants responded positively, particularly valuing the automatic scheduling of exercise appointments alongside other treatments. This study builds from previous investigations that showed inconsistencies in previous exercise referral models^15^ and that tailored exercise discussion was desired but missing from care.^21^

The referral workflow design made use of an implementation facilitator identified in previous research^10,22^ and reinforces the importance of patient care journey being fully integrated to normalise exercise as part of standard care. System-level changes, such as opt-out referral models, have been shown to significantly improve attendance.^12,13^ Our findings support this, demonstrating the reduced burden on patients to initiate their engagement with exercise. Instead, their attendance occurred seamlessly as part of their care pathway. Further, shifting the responsibility for initiating the referral away from the oncologist was well received by these participants, particularly when the oncologist reinforced the message about the importance of exercise. Beyond the automated appointment system, this study highlights the role of addressing misinformation and reframing of exercise from a discretionary activity to a medically endorsed component of cancer care. This finding aligns with previous qualitative research, where the importance of educating patients about the benefits of exercise has been shown to positively influence engagement.^23^ Importantly, participants also articulated that engaging in exercise provided a sense of control and agency during a period of vulnerability, underscoring the concurrent psychological benefits of exercise alongside the physical benefits.

For this program, the focus was on delivery of guideline-concordant care, i.e. ensuring all patients received initial exercise counselling, which is currently lacking.^24^ Resourcing was not available to deliver on-going exercise engagement within the organisation, which participants raised as an area for improvement. A broader challenge remains towards supporting patients with access to appropriate and sustained exercise services beyond the initial clinical setting, in both private and public care. Addressing this gap will require the establishment of partnerships with community-based exercise providers. These collaborations must be strategically developed, considering the specific needs, capacities, and constraints of community providers to support effective and sustainable long-term service delivery.

This study was conducted in a discrete private clinical setting characterised by strong institutional support for exercise integration, a collaborative relationship with the research team and high levels of patient engagement with exercise during their treatment. While this structure facilitated exercise delivery, the generalisability of the findings are reduced, as the level of infrastructure, clinician engagement, and organisational alignment observed is likely to exceed what is typically available in standard oncology care environments. The integration of EPs, structured appointments, and tailored counselling comes with substantial resourcing in delivery of exercise. Without organisational support, equitable access to exercise services will remain limited. Importantly, even within this supportive setting, challenges still emerged in implementing exercise as part of routine care, emphasising the need for continued focus on understanding *how* to deliver exercise for people living with and beyond cancer. For other settings looking to implement exercise, key features of the workflow that should be considered were opt-out referrals, consistent reinforcement of exercise by all providers, and the availability of appropriately qualified EPs with expertise in exercise oncology. Other limitations of this study include the structured nature of the focus group discussions, which were based on predefined topics. While this ensured coverage of key areas, it may have shaped participant responses and limited the emergence of unanticipated insights. The group format may also have constrained some individuals from sharing personal or divergent views, particularly if they felt uncomfortable expressing disagreement or vulnerability in a shared setting. Additionally, all participants voluntarily took part in the study, reflecting a possible bias toward interest in exercise from these discussions. Given the descriptive qualitative design and narrow, bounded focus of this study, meaning saturation was considered the most appropriate benchmark. The scope was deliberately limited to a single, well-defined referral process, and participants were purposively sampled for their direct experience. Consistency across the two focus groups further supports that sufficient meaning saturation was achieved.

## Conclusion

This study contributes to the growing body of research looking to understand patient perspectives into what helps facilitate exercise referrals during cancer treatment. Future research will explore the referral processes from the perspective of staff at the organisation, aiming to identify organisational and workflow factors that influence engagement of patients with exercise programs.

## Supplemental Material

Supplemental Material - “It Was Part of the Plan, so I Showed up”: An Exploration of Patient Experiences With an Embedded Exercise Referral Process During Cancer Treatment Supplemental Material for “It Was Part of the Plan, so I Showed up”: An Exploration of Patient Experiences With an Embedded Exercise Referral Process During Cancer Treatment by Mary A Kennedy, Jack Dalla Via, Kim Edmunds, Pam Eldridge, Yvonne Zissiadis, Lauren Fortington in Cancer Control.

## Data Availability

The data (transcripts) that support the findings of this study are not openly available due to reasons of sensitivity. Quotes are presented in the manuscript to support findings.*

## Appendix

### Abbreviations

CoLEC: co-located exercise service
RE-ACT: Referring to Exercise: Appointment, Counselling, Tailoring
EP: exercise physiologist
PSO: patient service officer.

## References

[1] Bai X-L Li Y Feng Z-F , et al. Impact of exercise on health outcomes in people with cancer: an umbrella review of systematic reviews and meta-analyses of randomised controlled trials. Br J Sports Med. 2025;59:1010-1020. doi:10.1136/bjsports-2024-109392. 40300838

[2] Campbell KL Winters-Stone KM Wiskemann J , et al. Exercise Guidelines for Cancer Survivors: Consensus Statement from International Multidisciplinary Roundtable. Med Sci Sports Exerc. 2019;51:2375-2390. doi:10.1249/MSS.0000000000002116. 31626055 PMC8576825

[3] Avancini A Borsati A Toniolo L , et al. Physical activity guidelines in oncology: A systematic review of the current recommendations. Crit Rev Oncol Hematol. 2025;210:104718. doi:10.1016/j.critrevonc.2025.104718. 40194715

[4] Cormie P Atkinson M Bucci L , et al. Clinical Oncology Society of Australia position statement on exercise in cancer care. Med J Aust. 2018;209:184-187. doi:10.5694/mja18.00199. 29719196

[5] Ligibel JA Bohlke K May AM , et al. Exercise, Diet, and Weight Management During Cancer Treatment: ASCO Guideline. J Clin Oncol Off J Am Soc Clin Oncol. 2022;40:2491-2507. doi:10.1200/JCO.22.00687. 35576506

[6] Schmitz KH Campbell AM Stuiver MM , et al. Exercise is medicine in oncology: Engaging clinicians to help patients move through cancer. CA Cancer J Clin. 2019;69:468-484. doi:10.3322/caac.21579. 31617590 PMC7896280

[7] Avancini A Pala V Trestini I , et al. Exercise Levels and Preferences in Cancer Patients: A Cross-Sectional Study. Int J Environ Res Public Health. 2020;17:5351. doi:10.3390/ijerph17155351. 32722265 PMC7432474

[8] Boyle T Vallance JK Ransom EK Lynch BM . How sedentary and physically active are breast cancer survivors, and which population subgroups have higher or lower levels of these behaviors? Support Care Cancer. 2016;24:2181-2190. doi:10.1007/s00520-015-3011-3. 26563180

[9] Cao C Patel AV Liu R Cao Y Friedenreich CM Yang L . Trends and cancer-specific patterns of physical activity, sleep duration, and daily sitting time among US cancer survivors, 1997-2018. JNCI J Natl Cancer Inst. 2023;115:1563–1575. doi: 10.1093/jnci/djad146 37527029 PMC10699842

[10] Kennedy MA Bayes S Newton RU , et al. Implementation barriers to integrating exercise as medicine in oncology: an ecological scoping review. J Cancer Surviv. 2022;16:865-881. doi:10.1007/s11764-021-01080-0. 34510366 PMC9300485

[11] Turner RR Arden MA Reale S , et al. The development of a theory and evidence-based intervention to aid implementation of exercise into the prostate cancer care pathway with a focus on healthcare professional behaviour, the STAMINA trial. BMC Health Serv Res. 2021;21:273. doi:10.1186/s12913-021-06266-x. 33766001 PMC7992804

[12] Caperchione CM English M Sharp P , et al. Exploring the practicality and acceptability of a brief exercise communication and clinician referral pathway in cancer care: a feasibility study. BMC Health Serv Res. 2023;23:1023. doi:10.1186/s12913-023-10003-x. 37740170 PMC10517509

[13] Finless A Bansal M Christensen T , et al. Exploring Healthcare Provider Experiences with the EXCEL Exercise Referral Pathway for Individuals Living with and Beyond Cancer. Curr Oncol. 2025;32:181. doi:10.3390/curroncol32030181. 40136385 PMC11941420

[14] Kennedy MA Bayes S Galvão DA , et al. If you build it, will they come? Evaluation of a co‐located exercise clinic and cancer treatment centre using the RE‐AIM framework. Eur J Cancer Care (Engl). 2020;29:e13251. doi:10.1111/ecc.13251. 32495410

[15] Kennedy MA Bayes S Newton RU , et al. Building the plane while it’s flying: implementation lessons from integrating a co-located exercise clinic into oncology care. BMC Health Serv Res. 2022;22:1235. doi:10.1186/s12913-022-08607-w. 36203189 PMC9535901

[16] Sandelowski M . Whatever happened to qualitative description? Res Nurs Health. 2000;23:334-340. doi:10.1002/1098-240x(200008)23:4<334::aid-nur9>3.0.co;2-g. 10940958

[17] Sundler AJ Lindberg E Nilsson C Palmér L . Qualitative thematic analysis based on descriptive phenomenology. Nurs Open. 2019;6:733-739. doi:10.1002/nop2.275. 31367394 PMC6650661

[18] Hitch D . Artificial Intelligence Augmented Qualitative Analysis: The Way of the Future? Qual Health Res. 2024;34:595-606. doi:10.1177/10497323231217392. 38064244 PMC11103925

[19] Czosnek L Richards J Zopf E Cormie P Rosenbaum S Rankin NM . Exercise interventions for people diagnosed with cancer: a systematic review of implementation outcomes. BMC Cancer. 2021;21:643. doi:10.1186/s12885-021-08196-7. 34053445 PMC8166065

[20] Ezenwankwo EF Nnate DA Usoro GD , et al. A scoping review examining the integration of exercise services in clinical oncology settings. BMC Health Serv Res. 2022;22:236. doi:10.1186/s12913-022-07598-y. 35189864 PMC8859567

[21] Dalla Via J Andrew CR Baguley BJ , et al. Exercise and diet support in breast and prostate cancer survivors: findings from focus groups. Support Care Cancer. 2024;32:440. doi:10.1007/s00520-024-08652-7. 38888665 PMC11189317

[22] Cantwell M Walsh D Furlong B , et al. Healthcare professionals’ knowledge and practice of physical activity promotion in cancer care: Challenges and solutions. Eur J Cancer Care (Engl). 2018;27:e12795. doi:10.1111/ecc.12795. 29193416

[23] Finch A Benham A . Patient attitudes and experiences towards exercise during oncological treatment. A qualitative systematic review. Support Care Cancer. 2024;32:509. doi:10.1007/s00520-024-08649-2. 38992238 PMC11239782

[24] Dalla Via J Cehic F Peddle-McIntyre CJ , et al. Translating advocacy into action: exploring oncology healthcare professionals’ awareness and use of the Clinical Oncology Society of Australia position statement on exercise in cancer care. Support Care Cancer Off J Multinatl Assoc Support Care Cancer. 2025;33:581. doi:10.1007/s00520-025-09633-0. PMC12167252 40515951

