# Supplementary material for: “It Was Part of the Plan, so I Showed up”: An Exploration of Patient Experiences With an Embedded Exercise Referral Process During Cancer Treatment
Source: Cancer Control. 2026 May 12;33:10732748261451084. doi: 10.1177/10732748261451084 (PMC13173343; doi:10.1177/10732748261451084)

Supplementary file 1: Demographic and physical activity questions.

Demographics (selected best option from choices, with other written if required)

- What is your gender? (e.g. male, female, other)
- Which of the following best describes your marital status? (e.g. married, widowed, etc)
- Which of the following best describes your ethnic background? (e.g. Aboriginal or Torres Strait Islander, Caucasian, Asian, etc)
- What is the highest educational level you have completed? (e.g. high school, trade certificate, bachelor's degree, etc)
- What is your usual major activity now? (e.g. employment, retired)
- Please select all the option(s) that best describe where your source of income came from in the last 10 years (e.g. professional work, labour work, retirement, etc)

Physical Activity

- Prior to your cancer treatment, do you think you regularly met the physical activity guidelines? (yes or no)
- What about your physical activity levels made you select this response? (open text)
- What motivated you to be regularly physically active? (open text)
- Why do you think you were not regularly physically active? (open text)
- Following diagnosis and during your cancer treatment, do you think you increased, maintained or decreased your physical activity levels? (open text)
- Why do you think this was? (open text)
- Since completing your cancer treatment (or since completing your primary cancer treatment if you are continuing hormone therapy), do you think you have increased, maintained or decreased your physical activity levels, as compared to your physical activity levels during treatment? (select increased, maintained or decreased)
- Why do you think this is? (open text)

Supplementary file 2: Guide used for focus g

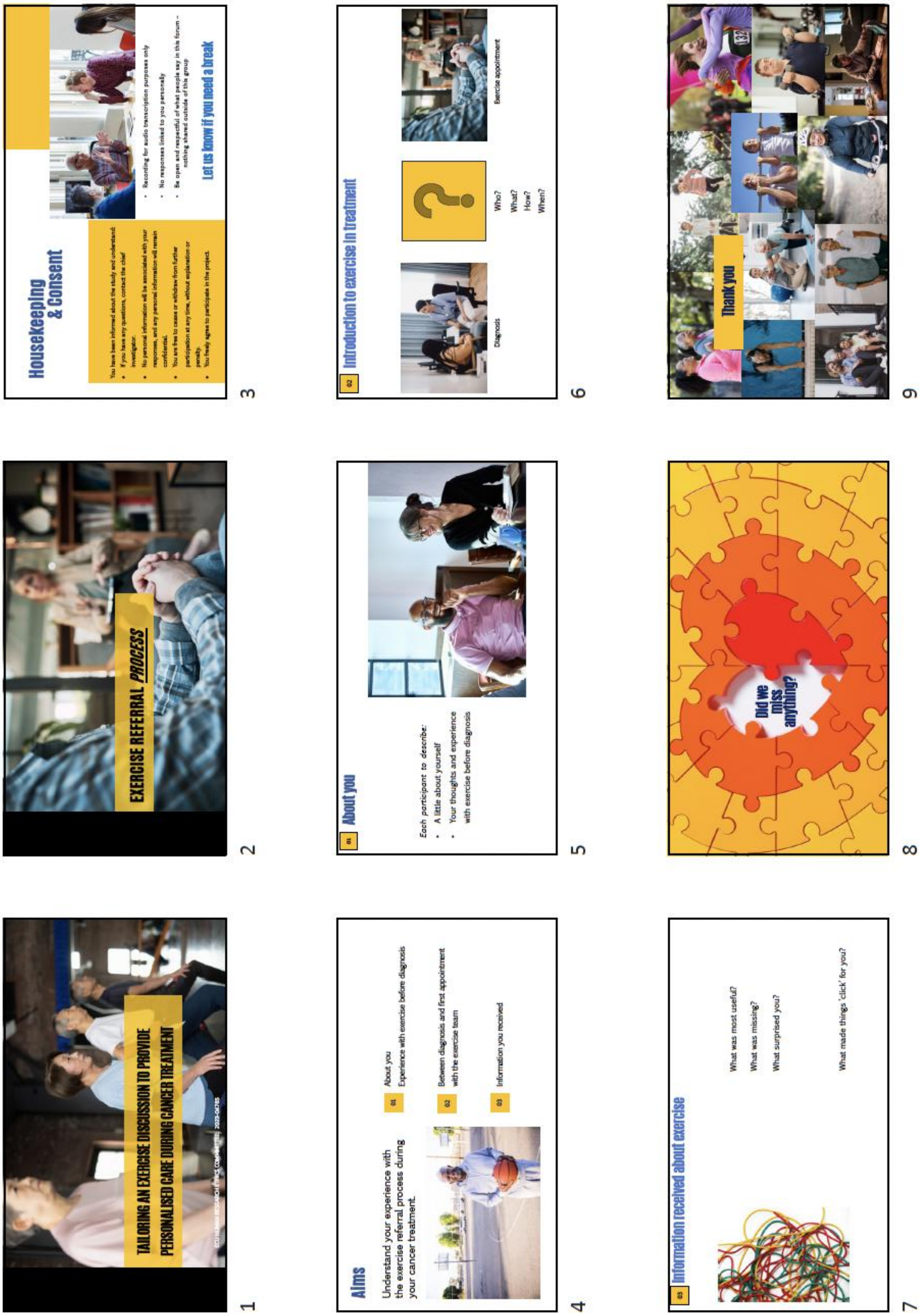

Supplement: Supplemental Material - “It Was Part of the Plan, so I Showed up”: An Exploration of Patient Experiences With an Embedded Exercise Referral Process During Cancer Treatment [file sj-pdf-1-ccx-10.1177_10732748261451084.pdf]
